# Gastric Myoelectric Activity in Patients With Metabolic Dysfunction-Associated Steatotic Liver Disease: A Case-Control Study

**DOI:** 10.7759/cureus.94830

**Published:** 2025-10-17

**Authors:** Gokul Shaji, Shanthi Vijayaraghavan

**Affiliations:** 1 Department of Hepatology, Sri Ramachandra Medical College, Chennai, IND

**Keywords:** electrogastrography, gastric motility, metabolic dysfunction-associated steatotic liver disease, myoelectric, water load standard test

## Abstract

Background

Metabolic dysfunction-associated steatotic liver disease (MASLD) is increasingly recognized for its extrahepatic manifestations, including gastrointestinal dysfunctions such as gastroparesis. This case-control study aimed to determine whether patients with MASLD exhibit gastric myoelectric abnormalities using electrogastrography (EGG).

Methodology

A case-control study was conducted with 16 MASLD patients and 16 healthy controls. Participants underwent EGG recordings in fasting and post-prandial states, including a water load standard test (WLST). Demographic data and gastric myoelectric parameters were collected and analyzed using paired Student’s t-tests and chi-square analysis.

Results

MASLD patients and controls were demographically well-matched. MASLD patients exhibited significantly higher water ingestion volumes during WLST (987.5 ± 222.5 mL vs. 656.3 ± 405.7 mL; p = 0.002), suggesting altered gastric accommodation. Trends toward hyponormal 3 cpm gastric myoelectric activity were observed in MASLD patients, though not statistically significant. Dysfunctional patterns related to interstitial cells of Cajal and antral pacemaker dysfunction were similarly distributed across both groups.

Conclusions

MASLD is associated with impaired gastric accommodation and possible myoelectric abnormalities, supporting its role as a multisystem disorder with gastrointestinal involvement. EGG may offer objective insights into gastric dysfunction in MASLD. Further large-scale studies are warranted to confirm these findings and integrate advanced motility testing.

## Introduction

Metabolic dysfunction-associated steatotic liver disease (MASLD), formerly known as non-alcoholic fatty liver disease, is a prevalent chronic liver condition characterized by excessive fat accumulation in the liver, not attributable to significant alcohol consumption [1]. Beyond its hepatic implications, MASLD is increasingly recognized as a multisystem disorder with a wide array of extrahepatic manifestations, significantly contributing to overall morbidity and mortality [2,3]. These systemic impacts extend to cardiovascular disease, type 2 diabetes mellitus, chronic kidney disease, and various gastrointestinal complications [4,5].

Among the gastrointestinal manifestations, gastroparesis, a syndrome defined by delayed gastric emptying in the absence of mechanical obstruction, is of particular interest in MASLD patients [6]. Symptoms commonly associated with gastroparesis include nausea, vomiting, early satiety, postprandial fullness, and abdominal pain, which can significantly impair quality of life [7]. While diabetes mellitus is a well-established cause of gastroparesis, the direct association between MASLD and gastric motility disorders, independent of diabetes, is an emerging area of research [8].

Electrogastrography (EGG) is a non-invasive technique used to record gastric myoelectrical activity, providing insights into the electrical control of gastric motility [9]. EGG can detect abnormalities in gastric slow waves, such as bradygastria (slow rhythm), tachygastria (fast rhythm), and dysrhythmias, which may correlate with symptoms of gastroparesis [10]. Given the systemic nature of MASLD and its potential influence on various physiological systems, investigating gastric myoelectric activity in this patient population is crucial for a comprehensive understanding of the disease’s pathophysiology and for developing targeted management strategies.

Previous studies have explored gastric motility in liver diseases, including cirrhosis, but specific research focusing on MASLD and its impact on gastric myoelectric activity is limited, particularly in the Indian context [11]. The current study aims to bridge this knowledge gap by evaluating gastric myoelectric abnormalities in MASLD patients using EGG, thereby contributing to a better understanding of the extrahepatic gastrointestinal manifestations of MASLD. We hypothesized that MASLD patients would demonstrate significant gastric myoelectric abnormalities compared to healthy controls. The objective of this study was to evaluate gastric myoelectric abnormalities in MASLD patients using EGG.

## Materials and methods

Study design and participants

This case-control study was conducted at Sri Ramachandra Institute of Higher Education and Research between September 2024 and February 2025. The study aimed to evaluate gastric myoelectric abnormalities in patients with MASLD. A total of 32 participants were enrolled, comprising 16 MASLD patients (cases) and 16 healthy controls.

Inclusion criteria

All inpatients and outpatients diagnosed with MASLD in the Department of Hepatology who provided informed consent were included as cases. Healthy adults aged 18 years or older were included as controls.

Exclusion criteria

Patients with any of the following conditions were excluded from the study: diabetes mellitus, neurological disease, current medication with prokinetic agents, infections, malignancies, myogenic diseases, thyroid disorder, or chronic liver disease. These exclusions were implemented to minimize confounding factors and ensure that observed gastric myoelectric abnormalities were primarily associated with MASLD.

Initial evaluation

Before EGG recordings, all participants underwent a comprehensive initial evaluation. This included a detailed history and physical examination to assess their overall health status and identify any pre-existing conditions. Specific attention was paid to a history of prior neurological disease, current drug intake (especially prokinetic agents), and any history of gastric surgery. Laboratory investigations were performed, including thyroid function test, complete blood count, fasting blood sugar, postprandial blood sugar, glycated hemoglobin (HbA1c) to rule out diabetes mellitus, liver function tests, lipid profile, international normalized ratio, and ultrasonography of the abdomen.

MASLD is defined by hepatic steatosis involving at least 5% of hepatocytes, detected by imaging or histology, in the presence of one or more cardiometabolic risk factors, namely, overweight or obesity (body mass index ≥25 kg/m²; ≥23 kg/m² for Asians, waist circumference ≥94 cm in men/≥80 cm in women for Europeans; ≥90 cm in men/≥ 80 cm in women for South Asians), type 2 diabetes mellitus (HbA1c ≥6.5%, FBS ≥126 mg/dL), dyslipidemia (triglycerides ≥150 mg/dL or high-density lipoprotein <39 mg/dL in men/<50 mg/dL in women), hypertension (blood pressure ≥130/85 mmHg), or dysglycemia (fasting glucose ≥100-125 mg/dL, HbA1c ≥5.7-6.4%) [1].

Electrogastrography procedure

Gastric myoelectric activity was measured using surface EGG. The procedure involved skin preparation; the abdominal skin over the epigastric region was shaved (if necessary) and thoroughly cleaned to ensure optimal electrode contact and minimize signal interference. Two electrodes were then placed to yield a bipolar EGG signal, capturing the electrical activity of the stomach. A third electrode was used as a reference electrode. The EGG signal was recorded using an ambulatory EGG recording unit, designed for continuous and stable data acquisition. The participants were positioned in a supine position and were instructed to remain as still as possible and avoid talking throughout the recording period. This was crucial to prevent motion artifacts that could compromise the quality of the EGG signal. All subjects fasted for at least eight hours before the study.

EGG recordings were performed in two phases, namely, a fasting state and a postprandial state. In the fasting state, a 10-minute recording was conducted while the subject was in a fasted state, which measures the resting motility pattern. During fasting, the stomach shows a regular but low-amplitude electrical rhythm. A 10-minute period provides enough data to confirm rhythm stability, reduction of transient artifacts (movement, respiration, noise). The 10-minute measurement also provides a reliable mean frequency and power value for comparison with post-meal activity. While the postprandial state (water load standard test, WLST) was done immediately following the fasting recording, subjects ingested a stimulation medium as part of the WLST. A 60-minute EGG recording was then performed to assess gastric myoelectric activity in response to the meal. Postprandial measurement of EGG is taken for 60 minutes because the postprandial response typically begins within 10-15 minutes and peaks by 30-60 minutes. This period also captures the most stable and sustained enhancement of gastric electrical activity. Some patients with delayed gastric response or gastroparesis show late or blunted changes, and a 60-minute postprandial measurement allows better detection. After the recording session, the EGG unit was connected to a computer, and the collected data were downloaded for subsequent analysis.

EGG provides a non-invasive method for assessing gastric myoelectrical activity and its modulation. It records the cutaneous potentials generated by the gastric slow waves originating from the interstitial cells of Cajal, which control the coordination and frequency of gastric contractions. Dominant frequency (DF) is a principal EGG parameter, which reflects the primary gastric rhythm, which normally ranges from 2.4 and 3.7 cycles per minute (cpm). Deviations below or above this range correspond to bradygastria and tachygastria, respectively, which are indicative of abnormal pacemaker activity. Another EGG parameter, dominant power or signal amplitude at the DF, represents the strength and synchrony of slow-wave propagation. Another parameter, postprandial-to-fasting power ratio, serves as a dynamic index of gastric motor responsiveness, with values greater than 1.0 indicating a normal postprandial enhancement in activity. There are other derived measures, such as the percentage distribution of slow-wave activity across frequency bands and the instability coefficient, which help quantify rhythm stability. Multichannel EGG allows calculation of a coupling index, reflecting the spatial coordination of slow waves between gastric regions. All these parameters collectively provide quantitative insights into the frequency, amplitude, and stability of gastric myoelectric activity. Deviations in these parameters, such as a blunted postprandial power response or increased dysrhythmic activity, can represent underlying autonomic or neuromuscular dysfunction, which links gastric myoelectrical abnormalities to impaired motility.

Statistical analysis

Statistical analysis was performed to compare variables between the MASLD and control groups. Independent Student’s t-test was used to compare continuous variables between MASLD and control groups, while chi-square analysis was applied for categorical variables. All quantitative values were represented as mean ± standard deviation. A p-value <0.05 was considered statistically significant.

Ethical considerations

The study protocol was approved by the Institutional Ethics Committee of Sri Ramachandra Institute of Higher Education and Research (approval number: CSP/MED/24 NOV/11/357). Written informed consent was obtained from all participants. The study aimed to identify the incidence of gastroparesis in MASLD patients, which could lead to earlier diagnosis and potential treatment, thereby offering a direct benefit to the participants. No significant ethical issues were foreseen or encountered during the study.

## Results

This study enrolled 32 participants, comprising 16 MASLD patients and 16 healthy controls. Baseline characteristics are shown in Table 1. Both MASLD patients and controls were well-matched in terms of age and gender distribution. The mean age of MASLD patients was 42.81 ± 9.89 years, and for controls, the mean age was 44.5 ± 12.12 years (t = 0.11, p = 0.910). Gender distribution also showed no significant difference: 11/16 (68.8%) males in MASLD vs. 12/16 (75.0%) in controls (χ² = 0.09, p = 1.000). This demographic matching helps minimize confounding effects in the subsequent analyses (Table 1).

**Table 1:** Baseline demographic characteristics of the study participants. Values expressed as mean ± standard deviation (SD) or number (N) with percentage (%). Statistical tests: independent Student’s t-test for continuous variables, chi-square test for categorical variables. t and χ² values are reported where applicable. A p-value <0.05 was considered statistically significant. MASLD = metabolic dysfunction-associated steatotic liver disease

Characteristics	MASLD (n = 16)	Control (n = 16)	Test statistic	P-value
Age (years)	42.81 ± 9.89	44.5 ± 12.12	t = 0.11	0.91
Gender				
Male	11 (68.8%)	12 (75.0%)	χ² = 0.00	1
Female	5 (31.2%)	4 (25.0%)		

**Figure 1:**
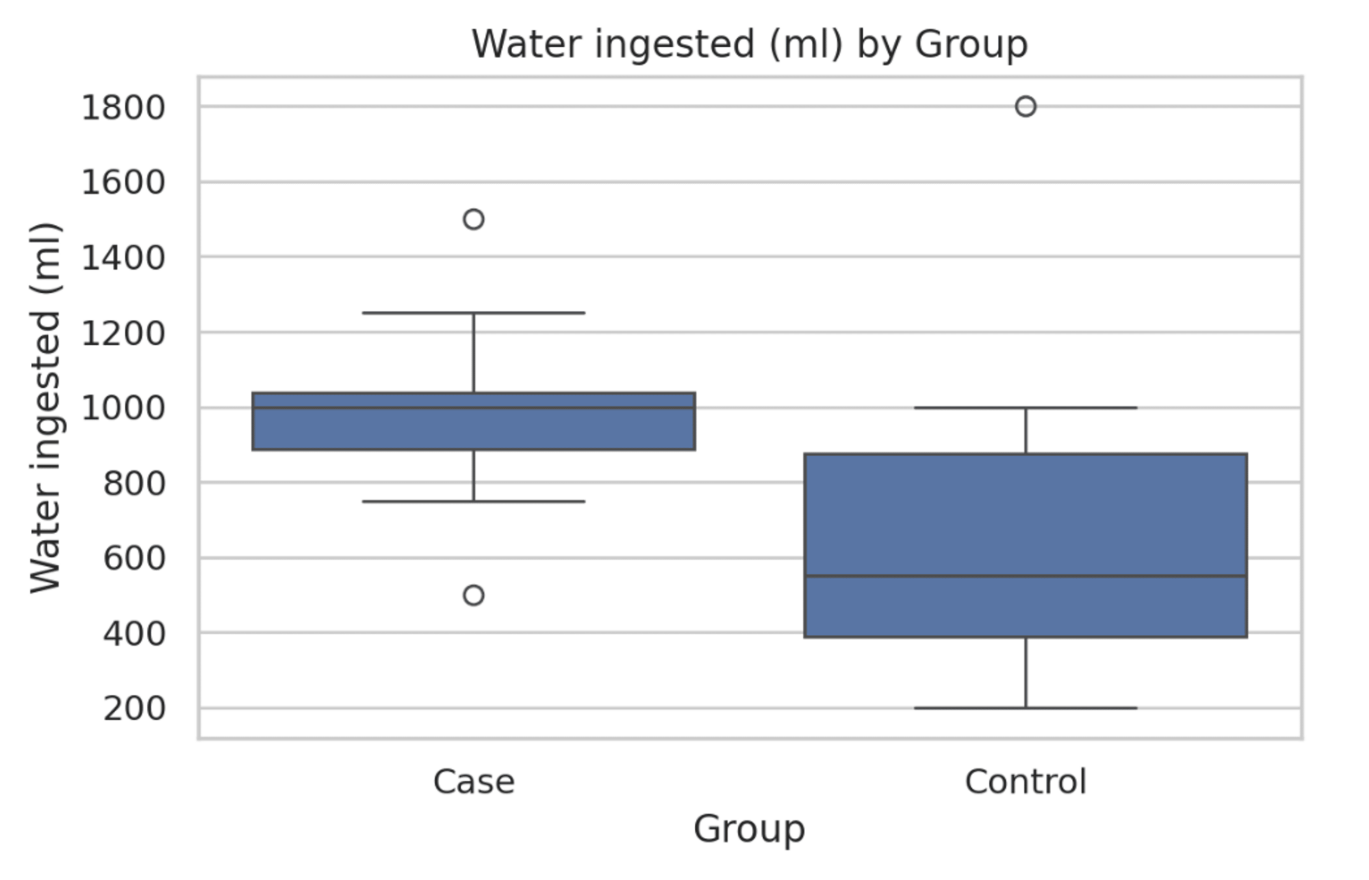
Comparison of water ingestion scores between the two groups. The bars represent mean water ingestion volumes (mL) during the WLST; error bars indicate SD. MASLD patients required significantly larger volumes compared to controls (t = 3.43, p = 0.002). A p-value <0.05 was considered statistically significant. WLST = water load standard test; MASLD = metabolic dysfunction-associated steatotic liver disease

**Figure 2:**
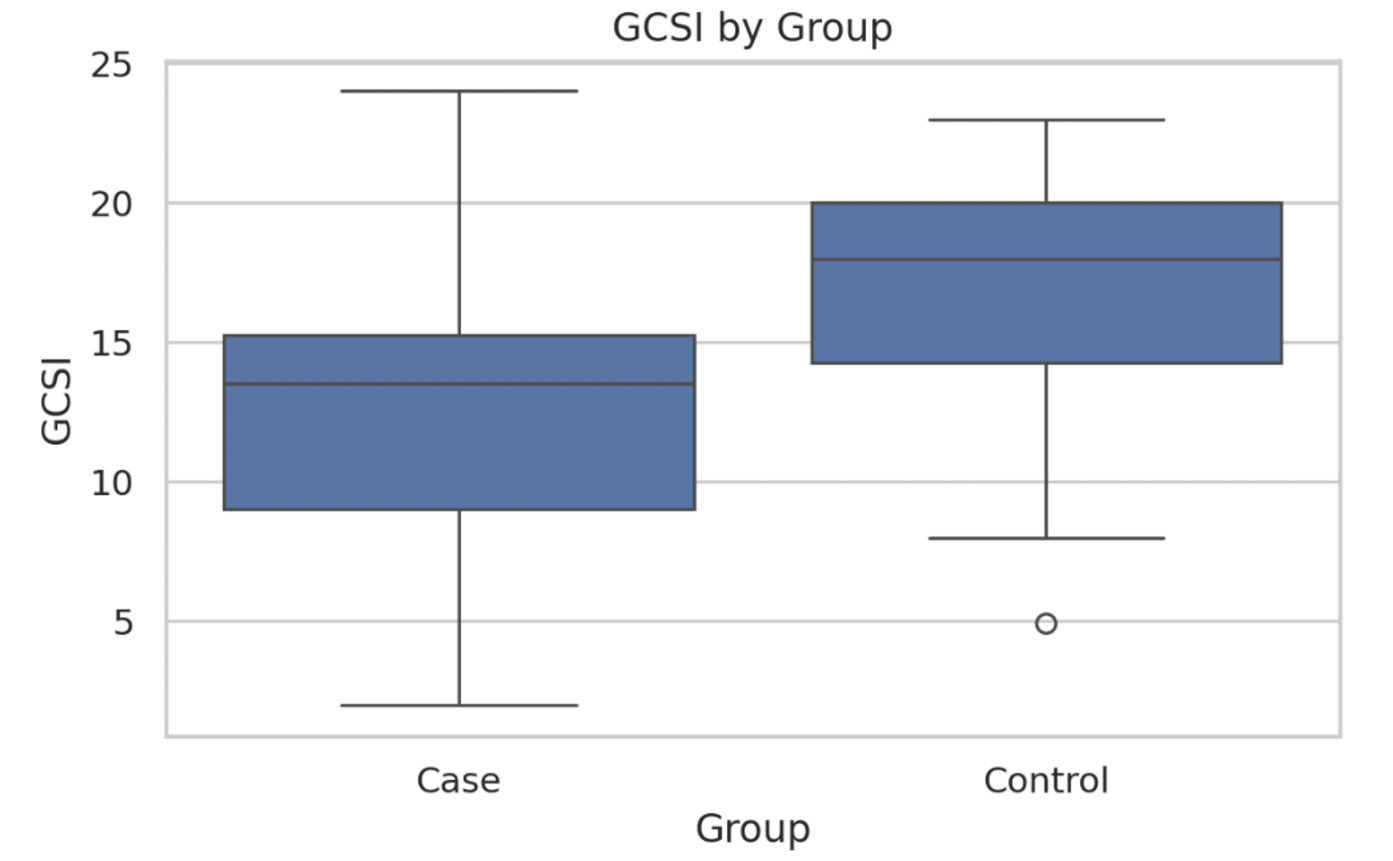
Comparison of GCSI scores between the two groups. The bars represent mean GCSI scores; error bars indicate SD. Comparison was performed using the independent Student’s t-test (t = 1.88, p = 0.071). A p-value <0.05 was considered statistically significant. GCSI = Gastroparesis Cardinal Symptom Index

**Figure 3:**
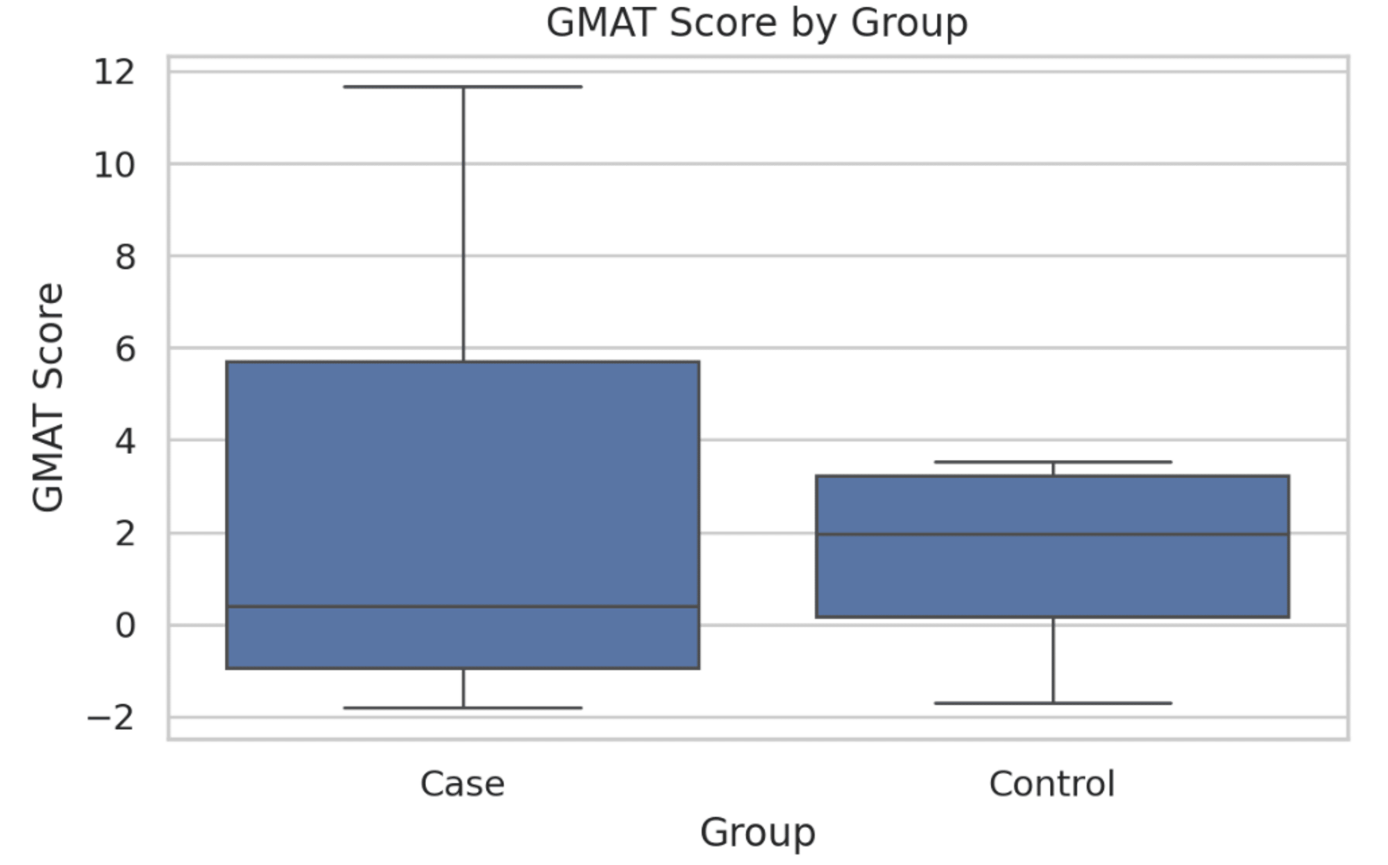
Comparison of GMAT scores between the two groups. The bars represent mean GMAT values; error bars indicate SD. Differences were not statistically significant (t = 0.54, p = 0.598). A p-value <0.05 was considered statistically significant. GMAT = gastric myoelectric activity time

Gastric emptying and myoelectric parameters are presented in Table 2. MASLD patients required significantly larger water ingestion volumes during WLST compared to controls (987.5 ± 222.5 mL vs. 656.3 ± 405.7 mL; t = 3.43, p = 0.002), suggesting altered gastric accommodation (Figure 1). The Gastroparesis Cardinal Symptom Index scores trended lower in MASLD patients (12.69 ± 5.80) compared to controls (16.12 ± 5.17) (t = 1.88, p = 0.071), but this difference did not reach statistical significance (p = 0.071) (Figure 2). Gastric myoelectric activity time showed no significant difference between groups (3.01 ± 5.65 vs. 1.44 ± 2.42) (t = 0.54, p = 0.598) (Table 2, Figure 3).

**Table 2:** Gastric emptying and myoelectric parameters. Values are expressed as mean ± SD. Comparisons were performed using an independent Student’s t-test. t values are reported. A p-value <0.05 was considered statistically significant. GCSI = Gastroparesis Cardinal Symptom Index; GMAT = gastric myoelectric activity time; MASLD = metabolic dysfunction-associated steatotic liver disease

Parameter	MASLD (n = 16)	Control (n = 16)	Test statistic	P-value
GCSI score	12.69 ± 5.80	16.12 ± 5.17	t = 1.88	0.071
Water ingestion (mL)	987.5 ± 222.5	656.3 ± 405.7	t = 3.43	0.002
GMAT	3.01 ± 5.65	1.44 ± 2.42	t = 0.54	0.598

Dysrhythmic gastric myoelectric responses are shown in Table 3. Abnormal gastric dysrhythmias were observed in both groups. MASLD patients showed hyponormal 3 cpm gastric myoelectric activity in 4/11 (36.4%) vs. 2/13 (15.4%) controls (χ² = 0.95, p = 0.33). Tachygastria was more frequent in controls (6/13; 46.2%) compared to MASLD patients (3/11; 27.3%). Though not statistically significant, these patterns suggest possible differences in the underlying myoelectric pathophysiology between MASLD and healthy individuals (Table 3).

**Table 3:** Dysrhythmic gastric myoelectric responses. Values are expressed as number (N) and percentage (%). Comparisons were performed using the chi-square test; χ² values are reported. A p-value <0.05 was considered statistically significant. GMA = gastric myoelectric activity; MASLD = metabolic dysfunction-associated steatotic liver disease

Dysrhythmic GMA response	MASLD (n = 11)	Control (n = 13)	Test statistic	P-value
Bradygastria	3 (27.3%)	3 (23.1%)	χ² = 1.84	0.6
Hyponormal 3 cpm GMA	4 (36.4%)	2 (15.4%)		
Mixed dysrhythmia	1 (9.1%)	2 (15.4%)		
Tachygastria	3 (27.3%)	6 (46.2%)		

Dysfunctional patterns based on interstitial cells of Cajal and antral pacemaker dysfunction are shown in Table 4. Antral pacemaker dysfunction was present in 3/16 (18.8%) of both MASLD and control groups (χ² = 0.00, p = 1.00). Interstitial cells of Cajal dysfunction was identical in both groups (11/16; 68.8%). This indicates that while MASLD patients demonstrate symptomatic differences, their underlying electrophysiological dysfunction mirrors that seen in the general population (Table 4).

**Table 4:** Dysfunctional patterns based on ICC and APD. Values are expressed as N (%). Comparisons were performed using the chi-square test; χ² values are reported. A p-value <0.05 was considered statistically significant. ICC = interstitial cells of Cajal; APD = antral pacemaker dysfunction; MASLD = metabolic dysfunction-associated steatotic liver disease

Dysfunction	MASLD (n = 16)	Control (n = 16)	Test statistic	P-value
APD	3 (18.8%)	3 (18.8%)	χ² = 0.00	1
ICC	11 (68.8%)	11 (68.8%)		
Normal	2 (12.5%)	2 (12.5%)		

## Discussion

This case-control study demonstrates that MASLD patients exhibit impaired gastric accommodation and trends toward altered gastric myoelectric activity compared to healthy controls, underscoring the systemic impact of MASLD beyond the liver.

One of the most notable findings of our study was the significantly larger water ingestion volumes required by MASLD patients during the WLST compared to controls (987.5 ± 222.5 mL vs. 656.3 ± 405.7 mL, p = 0.002). This suggests impaired gastric accommodation in MASLD patients, a physiological response where the stomach relaxes to accommodate ingested food without a significant rise in intragastric pressure. Altered gastric accommodation can lead to early satiety and postprandial fullness. This observation aligns with existing literature that highlights gastrointestinal dysmotility in various chronic liver diseases. Studies have shown that patients with cirrhosis, a more advanced stage of liver disease, often exhibit delayed gastric emptying and impaired gastric accommodation [11]. While the present study focuses on MASLD, a condition that may precede cirrhosis, the findings suggest that gastric physiological changes can occur even in earlier stages of liver metabolic dysfunction. A recent study by Wang et al. (2025) also utilized EGG to assess the relationship between MASLD and gastric electrical rhythm, indicating a strong association between MASLD and an increased incidence of diabetic gastroparesis, although their focus was on diabetic patients with MASLD [12]. The present study, by excluding diabetic patients, provides a clearer picture of MASLD-specific gastric dysfunction.

The EGG analysis revealed trends toward hyponormal 3 cpm gastric myoelectric activity in MASLD patients (36.4% vs. 15.4% in controls), while tachygastria was more frequent in controls (46.2% vs. 27.3%). Although these differences did not reach statistical significance, they suggest potential underlying myoelectric pathophysiology variations between MASLD patients and healthy individuals. The lack of statistical significance could be attributed to the small sample size, a common limitation in pilot studies. Larger studies are needed to confirm these trends and explore their clinical relevance. Research on gastric electrical rhythm in patients with metabolic dysfunction, including MASLD, is an evolving field. For example, a study by De et al. (2025) discussed the prevalence of MASLD in India, noting similar trends to global data, which underscores the relevance of findings of the present study to the Indian population [13]. While their study did not directly address EGG, it highlights the growing burden of MASLD in India, making research into its associated complications, such as gastric dysmotility, particularly pertinent.

Furthermore, the present study found similar distributions of dysfunctional patterns related to interstitial cells of Cajal and antral pacemaker dysfunction across both MASLD and control groups. This suggests that the fundamental electrophysiological machinery responsible for gastric rhythm generation (interstitial cells of Cajal) and coordination (antral pacemaker dysfunction) may not be uniquely or significantly impaired in MASLD patients compared to the general population. Instead, the observed functional differences, such as altered gastric accommodation, might stem from other factors, such as autonomic neuropathy, inflammatory processes, or altered gut-brain axis signaling, which are known to be influenced by metabolic dysregulation in MASLD [3]. This finding is crucial as it directs future research toward exploring these alternative pathways rather than solely focusing on primary myoelectric abnormalities in interstitial cells of Cajal and antral pacemaker dysfunction.

Comparing findings of the present study with other studies, the association between MASLD and gastrointestinal symptoms is increasingly recognized. Another Indian study by Misra et al. provided consensus guidelines for the diagnosis and management of MASLD in adult Asian Indians with type 2 diabetes, emphasizing the unique characteristics of MASLD in this population [14]. Although the present study excluded diabetic patients, the broader context of MASLD prevalence and management in India reinforces the importance of understanding its diverse manifestations. Hence, screening for gastric dysfunction in MASLD may allow earlier identification of extrahepatic complications and guide patient management.

Limitations

This study has several limitations. First, the small sample size (n = 32) limited statistical power and restricted generalizability. Second, as a single-center study, the external validity of our findings is limited. Third, although EGG is a non-invasive tool, it has lower sensitivity than gastric emptying scintigraphy, potentially underestimating gastric dysfunction. Finally, reliance on subjective symptom reporting likely introduced recall bias.

Recommendations

Future research should focus on larger multicenter studies to validate these preliminary findings and improve statistical power. Integration of advanced motility tests, such as wireless motility capsules or gastric emptying scintigraphy with EGG, would provide a more comprehensive evaluation of gastric function. Stratification of MASLD patients by disease severity (e.g., fibrosis or metabolic dysfunction-associated steatohepatitis status) may clarify specific dysfunction patterns. Longitudinal follow-up studies would provide further insight into the gastric myoelectric changes as MASLD progresses with disease severity. Finally, routine screening for gastrointestinal symptoms in MASLD clinics is recommended to facilitate early detection and management.

## Conclusions

MASLD should be recognized as a multisystem disease. Our findings of impaired gastric accommodation and possible myoelectric abnormalities, even in non-diabetic patients, highlight the need to include gastric function assessment in MASLD management. Future research building upon these findings will be essential to fully elucidate the complex interplay between MASLD and gastric motility, ultimately leading to improved diagnostic tools and therapeutic strategies.
